# Therapeutic Potential of Food-Derived Rutin Phytosome Nanoparticles: Anti-Tumor, Antioxidant, and Anti-Inflammatory Activity in Ehrlich Ascites Carcinoma

**DOI:** 10.3390/ph18091410

**Published:** 2025-09-19

**Authors:** M. Alfawaz, Ekramy M. Elmorsy, Alaa Samy, Ahmed S. Shams, Mai A. Salem, Aly A. M. Shaalan, Manal S. Fawzy, Nora Hosny

**Affiliations:** 1 Department of Medical Laboratory Technology, College of Applied Medical Sciences, Northern Border University, Arar 91431, Saudi Arabia; mohammed.alfawaz@nbu.edu.sa; 2 Center for Health Research, Northern Border University, Arar 73213, Saudi Arabia; ekramy.elmorsy@nbu.edu.sa; 3 Department of Forensic Medicine and Clinical Toxicology, Faculty of Medicine, Mansoura University, Mansoura 35516, Egypt; 4 Department of Surgery, Anaesthesiology and Radiology, Faculty of Veterinary Medicine, Mansoura University, Mansoura 35516, Egypt; alaasamy@mans.edu.eg; 5 Department of Human Anatomy and Embryology, Faculty of Medicine, Suez Canal University, Ismailia 41522, Egypt; ashams@umn.edu; 6 Department of Medical Biochemistry, Faculty of Medicine, Mansoura University, Mansoura 35516, Egypt; maisalem@mans.edu.eg; 7 Department of Anatomy, Faculty of Medicine, Jazan University, Jazan 45142, Saudi Arabia; ashaalan@jazanu.edu.sa; 8 Department of Histology, Faculty of Medicine, Suez Canal University, Ismailia 41522, Egypt; 9 Department of Medical Biochemistry and Molecular Biology, Faculty of Medicine, Suez Canal University, Ismailia 41522, Egypt; nora_hosny@med.suez.edu.eg

**Keywords:** rutin phytosome nanoparticles, Ehrlich ascites carcinoma, anti-tumor activity, apoptosis, antioxidant activity

## Abstract

**Background/Objectives:** Rutin (RT), a promising bioflavonoid, faces clinical limitations due to its poor solubility and bioavailability. In this study, we formulated RT-loaded phytosome nanoparticles (RT-PNPs) via thin-layer hydration and characterized their morphology, size distribution, and zeta potential. **Methods:** We established a mouse model of Ehrlich ascites carcinoma (EAC), randomly allocating ninety female Swiss albino mice into six groups: untreated controls, RT-treated, RT-PNP-treated, EAC, EAC + RT, and EAC + RT-PNPs. Tumor induction and treatment protocols were controlled, with the oral administration of 25 mg/kg/day of RT or RT-PNPs for 20 days. We comprehensively assessed survival, body weight, ascitic fluid/tumor volume, and cell viability and performed detailed hematological, serum biochemical, and tumor marker analyses. Multiorgan (liver and kidney) function and redox homeostasis were evaluated through enzymatic assays for SOD, CAT, GSH-Px, and GSH, as well as lipid peroxidation assessment. Proinflammatory cytokines and tumor markers (AFP, CEA, CA19-9, CA125, and CA15-3) were quantified via ELISA. **Results:** Gene expression profiling (*TP53*, *Bax*, and *Bcl-2*) and flow cytometry (p53 and Ki-67) elucidated the modulation of apoptosis. Histopathological scoring documented organ protection, while advanced multivariate (heatmap and principal component) analyses revealed distinct treatment clusterings. The RT-PNPs demonstrated potent anti-tumor, antioxidant, anti-inflammatory, and apoptosis-inducing effects, outperforming free RT in restoring physiological markers and tissue integrity. **Conclusions:** The current results underscore the potential of RT-PNPs as a multifaceted therapeutic approach to EAC, leveraging nanoparticle technology to optimize efficacy and systemic protection.

## 1. Introduction

Cancer remains a leading cause of mortality worldwide and is often incurable despite significant advances in radiation therapy, surgical techniques, and systemic treatments [1]. While conventional chemotherapy plays a crucial role in cancer management, its effectiveness is limited by poor selectivity for tumor cells, considerable toxicity, and the emergence of drug resistance [2]. Synthetic chemotherapeutic agents are often accompanied by a range of side effects, including gastrointestinal and renal disturbances, fatigue, bone marrow suppression, and the eventual development of resistance in cancer cells [3]. Consequently, cancer research has increasingly focused on developing new therapeutic strategies that offer high efficacy with minimal toxicity [4]. Free radicals play a key role in the onset of cancer, allergies, autoimmune disorders, and neurodegenerative diseases. The body is equipped with a complex system of endogenous antioxidant enzymes to combat oxidative stress. For decades, natural products have served as essential sources of effective, low-toxicity drugs [5,6].

**Figure 1:**
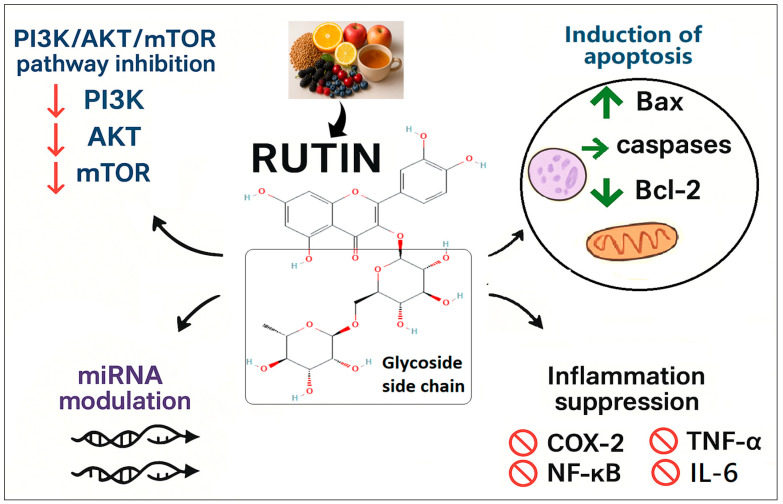
Proposed mechanisms of the anti-cancer effect of Rutin as reported in the literature. PI3K/AKT/mTOR: phosphoinositide 3 kinase/Akt/mammalian target of Rapamycin, miRNA: microRNA, Bax: Bcl-2–associated X protein, Bcl-2: B-cell lymphoma-2, COX-2: Cyclooxygenase-2, TNF-α: Tumour necrosis factor-alpha, NF-κB: Nuclear factor-kappa B, IL-6: Interleukin-6.

In numerous studies, Rutin (RT), a bioflavonoid compound (Chemical identifier; CID: 5280805), has been shown to possess potent anti-cancer, antioxidant, and anti-inflammatory properties [7,8,9,10]. RT is a glycoside derivative of the polyphenol quercetin. The presence of a disaccharide side chain enhances the solubility and bioavailability of quercetin compared to quercetin alone (Figure 1) [11]. This structural distinction also contributes to its more substantial antioxidant potential. Mechanistically, RT mediates its anti-cancer effects by modulating the PI3K/AKT/mTOR signaling pathway, thereby inducing apoptosis and inhibiting tumor proliferation [12]. It inhibits the phosphorylation of glycogen synthase kinase-3 beta (GSK-3β) and AKT, leading to the upregulation of tumor suppressor proteins [13]. Additionally, it has been shown to modulate microRNAs (miRNAs), which are crucial post-transcriptional regulators involved in cancer development. Specifically, RT can modulate the expression of both tumor-suppressive and oncogenic miRNAs, ultimately limiting the proliferation of cancer cells [14]. RT also exhibits strong anti-cancer potential by targeting both apoptosis and inflammation, which is a well-known contributor to tumorigenesis, by inhibiting key signaling molecules, including cyclooxygenase-2 (COX-2) and nuclear factor kappa B (NF-κB), and by suppressing proinflammatory cytokines such as interleukin-6 (IL-6) and tumor necrosis factor-α (TNF-α) [15]. RT concurrently induces apoptosis in cancer cells by modulating both extrinsic and intrinsic apoptotic pathways. It downregulates anti-apoptotic proteins such as Bcl-2 while upregulating pro-apoptotic proteins like Bax and caspases [16]. This dual mechanism underscores RT’s potential as a promising natural anti-cancer agent.

Although RT possesses great therapeutic potential, its clinical utility is restricted due to chemical instability and poor water solubility, which significantly limit its bioavailability [17]. The use of nanocarriers is a promising strategy to overcome these issues and enhance its antioxidant effects. To ensure successful and targeted delivery, it is vital to improve its bioavailability without compromising its pharmacological properties. Phytosomes represent a novel drug delivery platform that employs a phospholipid bilayer to form vesicles capable of entrapping both hydrophobic and hydrophilic molecules. This system improves stability, solubility, and membrane permeability by reducing the interfacial tension between poorly soluble agents and solvents [18]. Despite increasing attention to the pharmacological properties of RT, in vivo investigations using the Ehrlich ascites carcinoma (EAC) model are still limited. To the authors’ knowledge, this study is the first to evaluate RT-loaded nano-phytosomes (RT-PNPs) in this model, focusing on their antioxidant, anti-inflammatory, and apoptosis-inducing capabilities.

## 2. Results

### 2.1. Characterization of Rutin-Loaded Nano-Phytosomes (RT-PNPs)

**Figure 2:**
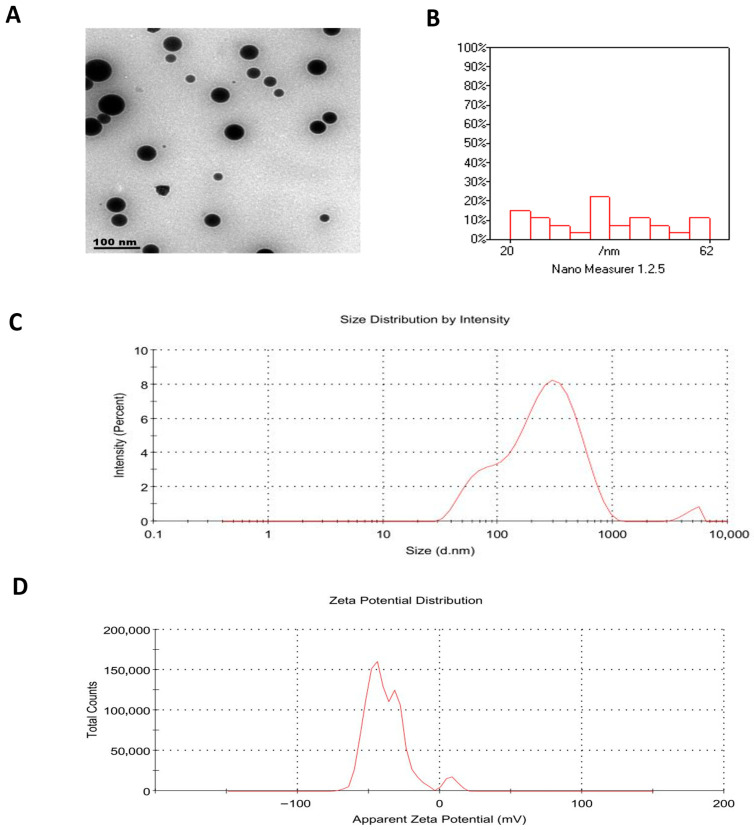
Characterization of Rutin-Loaded Phytosome Nanoparticles (RT-PNPs). (**A**) The spherical morphology by the transmission electron microscopy (TEM), (**B**) The particle size distribution ranging from 20 to 62 nm, (**C**) The volume-based size distribution, and zeta potential profile, (**D**) the measured values of 185 nm for Z-average size, 0.392 for PDI, and −37 mV for zeta potential.

Morphological examination by Transmission electron microscopy (TEM) confirmed that the RT-PNPs were spherical and exhibited uniformity in structure (Figure 2A), with particle sizes measured between 60 and 62 nm (Figure 2B). In comparison, dynamic light scattering (DLS) measurements indicated a larger hydrodynamic diameter (185 nm), likely due to interactions with the dispersion medium. As shown in Table 1, the particle sizes of the prepared formulations ranged from 185 to 260 nm, with higher PC: RT ratios and moderate cholesterol content. The polydispersity index values fell within the range of 0.39–0.47 (Figure 2C), reflecting acceptable uniformity. Furthermore, the zeta potential increased with the addition of phosphatidylcholine and moderate cholesterol, reaching −37.0 mV in F5 (Figure 2D), indicating enhanced colloidal stability. Among the tested formulations, encapsulation efficiency ranged from 66.3% (F1) to 84.62% (F5), with F5 exhibiting the superior drug loading. Given its favorable particle size, poly-dispersity index, zeta potential, and encapsulation performance, formulation F5 (PC: RT 1:2, 5% cholesterol) was selected as the optimized RT-PNPs.

**Figure 3:**
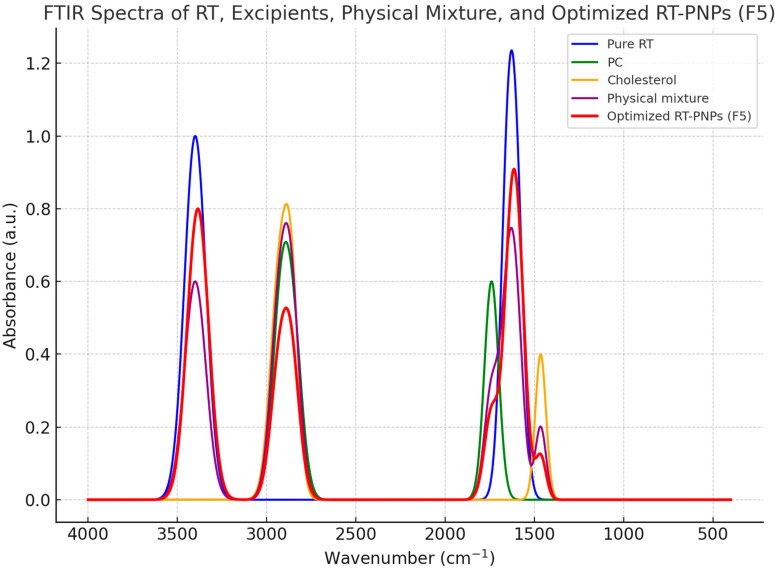
FTIR spectra of Rutin (RT), phosphatidylcholine (PC), cholesterol, physical mixture, and optimized RT-PNPs (F5). The major rutin absorption bands are conserved in the RT-PNPs spectrum with minor shifts and decreased intensity, suggesting non-covalent interactions and confirming component compatibility.

Fourier Transform Infrared (FTIR) spectroscopic analysis was performed to confirm the chemical compatibility and molecular interactions between rutin, the excipients, physical mixture, and the optimized RT-PNPs formulation (Figure 3). Pure RT displayed typical absorption bands approximately at 3400 cm^−1^ (O–H stretching), 1600 cm^−1^ (aromatic C=C stretching), and 1650 cm^−1^ (C=O stretching). The individual excipients (PC and cholesterol) demonstrated their specific characteristic peaks. In the physical mixture and optimized RT-PNPs spectrum, the primary rutin bands were retained; however, minor shifts in vibrational frequencies and reduced intensities were observed compared to free RT. These modifications, particularly slight displacement and attenuation of the O–H and C=O signals, reflect non-covalent interactions, mainly hydrogen bonding, between rutin and the lipid matrix. Notably, there were no novel peaks or marked peak broadening, indicating an absence of chemical incompatibility. These FTIR results confirm the stable encapsulation and compatibility of RT in the phytosomal structure.

### 2.2. Changes in Cell Volume, Viability, Body Weight, and the Mean Survival Time

**Figure 4:**
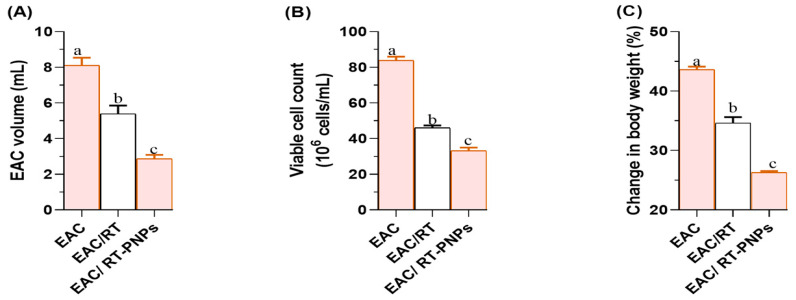
Changes in cell volume (**A**), viability (**B**), and body weight (**C**) in Ehrlich Ascites Carcinoma (EAC)-bearing mice treated with Rutin-loaded nano-phytosome and raw Rutin (*n* = 15 for each group). Values are presented as mean ± SE. One-way ANOVA with Tukey’s post hoc test was applied. Within the same row, values bearing different superscript letters (a, b, c) differ significantly at *p* < 0.05.

The mice inoculated with EAC exhibited a significant increase in the volume of ascitic fluid compared to those treated with either crude RT or rutin-loaded nano-phytosomes (RT-PNPs), with the reduction being significantly greater in the RT-PNP group than in the RT-treated group (*p* < 0.05; Figure 4A). Additionally, treatment with either RT or RT-PNPs following EAC injection significantly reduced cell viability, with a more pronounced decrease observed in the RT-PNP group compared to the crude RT group (*p* < 0.05; Figure 4B). Furthermore, the most significant increase in body weight was observed in untreated EAC-bearing mice, while treatment with RT-PNPs significantly reduced body weight gain, thereby demonstrating a greater effect than crude RT treatment (*p* < 0.05; Figure 4C). The estimated mean survival time was 9 days for untreated EAC-treated mice, which increased to 14 days for the RT-treated mice and 16.5 days for the RT-PNP-treated mice.

### 2.3. Hematology Parameters

Table 2 illustrates the hematological changes observed following treatment with either crude RT or RT-PNPs in EAC-bearing mice. The induction of EAC resulted in a significant reduction in red blood cell (RBC) count, platelet count (PLT), hemoglobin (Hb) concentration, and packed cell volume (PCV) compared to the control group. Notably, treatment with RT or RT-PNPs significantly improved these hematological parameters, with RT-PNPs demonstrating greater efficacy than RT in all parameters except Hb levels, for which the difference was not statistically significant. Both treatments effectively restored RBC count, PLT, and PCV values to those of the negative control. In contrast, erythrocyte indices, including mean corpuscular volume (MCV), mean corpuscular hemoglobin (MCH), and mean corpuscular hemoglobin concentration (MCHC), remained statistically unchanged across all treated groups. Moreover, white blood cell (WBC) counts were significantly elevated in EAC-bearing mice and significantly reduced following treatment with either RT or RT-PNPs, with no significant differences observed between the treated groups and the controls.

### 2.4. Blood Chemistry

Table 3 presents the changes in blood chemistry following treatment with either crude RT or RT-PNPs in mice bearing EAC. In this study, the levels of total protein and its fractions (albumin and globulin) were significantly reduced in EAC-treated mice compared to the control group (*p* < 0.05). Treatment with RT-PNPs significantly restored these levels, thus bringing total protein and globulin values close to those of the control group. Concerning lipid metabolism, total cholesterol and triglyceride levels were significantly elevated in EAC-bearing mice. However, they were still markedly reduced following treatment with either RT or RT-PNPs, with the EAC/RT-PNP group showing greater efficacy than the EAC/RT group. In terms of kidney function, serum creatinine and urea levels were significantly increased in EAC-bearing mice. Both parameters were significantly decreased following treatment with RT or RT-PNPs. Urea levels were substantially lower in the EAC/RT-PNP group compared to the EAC/RT group, while creatinine levels showed no significant difference between the two treated groups. The levels of liver function enzymes (AST, ALT, and ALP) were significantly elevated in EAC-treated mice but were significantly reduced following treatment with either RT or RT-PNPs. Enzyme levels were lower in the EAC/RT-PNP group compared to the EAC/RT group, with no significant differences observed between either of the treated groups and the negative control group.

### 2.5. Tumor-Associated Biomarkers

**Figure 5:**
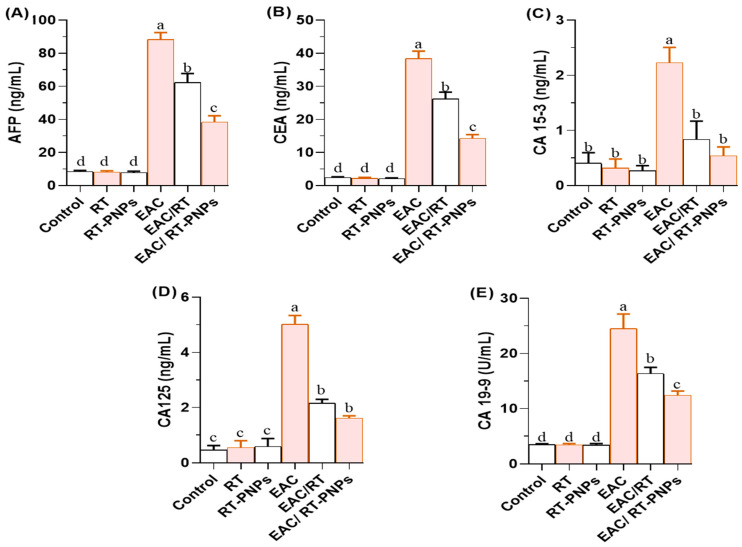
Changes in tumor-associated biomarkers in Ehrlich Ascites Carcinoma (EAC)-bearing mice treated with Rutin-loaded nano-phytosome and raw Rutin. (**A**) AFB: Alpha-Fetoprotein (**B**) CEA: Carcinoembryonic Antigen; (**C**) CA 15-3: Cancer Antigen 15-3; (**D**) CA 125: Cancer Antigen 125; (**E**) CA 19-9: Carbohydrate Antigen 19-9. RT: Rutin (25 mg/kg body weight); RT-PNPs: Rutin-loaded nano-phytosomes (25 mg/kg body weight); EAC: Inoculated with Ehrlich Ascites Carcinoma (EAC) cells (0.2 mL); EAC/RT: Rutin (25 mg/kg body weight) + EAC cells (0.2 mL); EAC/RT-PNPs: Rutin-loaded nano-phytosomes (25 mg/kg body weight) + EAC cells (0.2 mL) (*n* = 15 for each group. Values are presented as mean ± SE. One-way ANOVA with Tukey’s post hoc test was applied. Within the same row, values bearing different superscript letters (a, b, c, d) differ significantly at *p* < 0.05.

Figure 5A–E demonstrates that the levels of all examined tumor biomarkers were significantly elevated in EAC-treated mice, including alpha-fetoprotein (AFP), carcinoembryonic antigen (CEA), cancer antigen 15-3 (CA15-3), cancer antigen 125 (CA125), and carbohydrate antigen 19-9 (CA19-9). Treatment with either crude RT or RT-PNPs significantly reduced these levels. The EAC/RT-PNP group showed considerably lower levels than the EAC/RT group for AFP (Figure 5A), CEA (Figure 5B), and CA19-9 (Figure 5E). At the same time, no significant differences were observed between the two treated groups for CA15-3 (Figure 5C) and CA125 (Figure 5D). Additionally, CA15-3 levels in the EAC/RT-PNP group did not differ significantly from those in the negative control group (*p* > 0.05).

### 2.6. Redox Status

**Figure 6:**
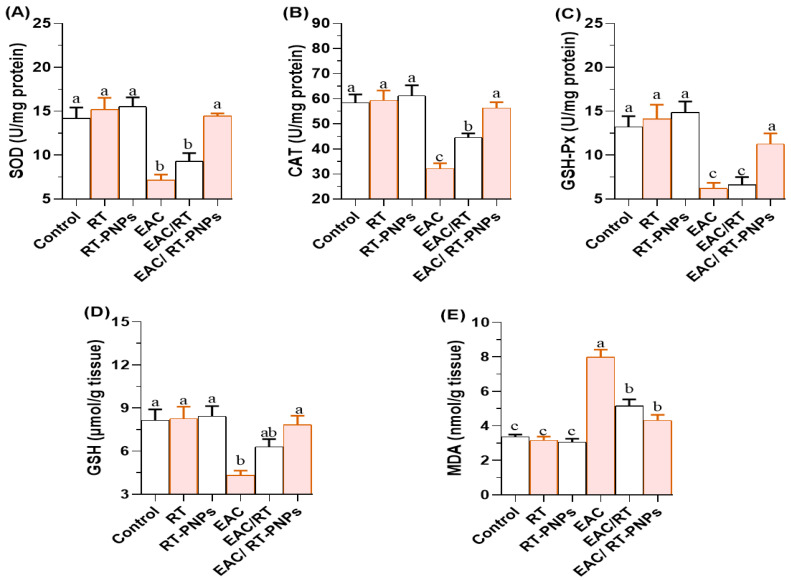
Changes in hepatic redox status in Ehrlich Ascites Carcinoma (EAC)-bearing mice treated with Rutin-loaded nano-phytosome and raw Rutin. (**A**) SOD: superoxide dismutase; (**B**) CAT: catalase; (**C**) GSH-Px: glutathione peroxidase; (**D**) GSH: reduced glutathione; (**E**) MDA: malondialdehyde. RT: Rutin (25 mg/kg body weight); RT-PNPs: Rutin-loaded nano-phytosomes (25 mg/kg body weight); EAC: Inoculated with Ehrlich Ascites Carcinoma (EAC) cells (0.2 mL); EAC/RT: Rutin (25 mg/kg body weight) + EAC cells (0.2 mL); EAC/RT-PNPs: Rutin-loaded nano-phytosomes (25 mg/kg body weight) + EAC cells (0.2 mL) (*n* = 15 for each group). Values are presented as mean ± SE. One-way ANOVA with Tukey’s post hoc test was applied. Within the same row, values bearing different superscript letters (a, b, c) differ significantly at *p* < 0.05.

**Figure 7:**
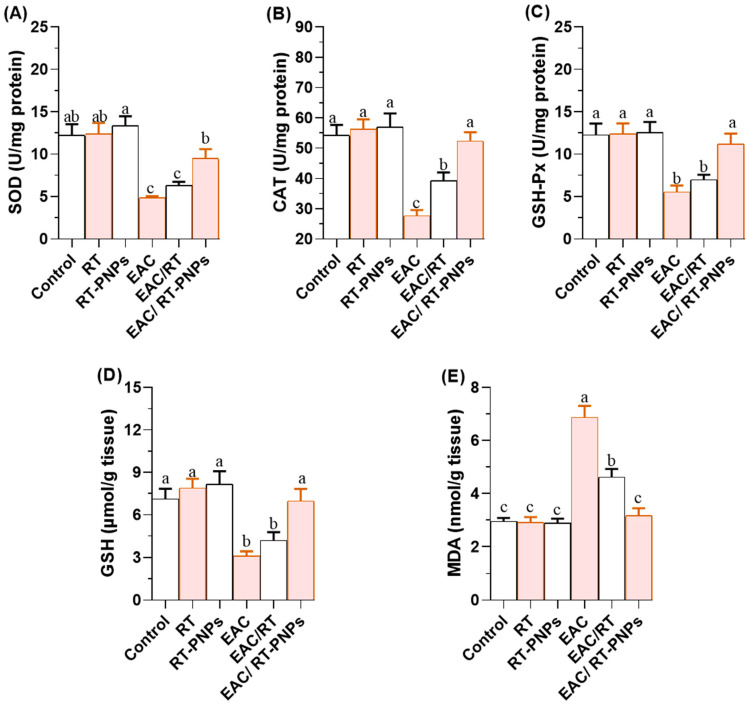
Changes in renal redox status in Ehrlich Ascites Carcinoma (EAC)-bearing mice treated with Rutin-loaded nano-phytosome and raw Rutin. (**A**) SOD: superoxide dismutase; (**B**) CAT: catalase; (**C**) GSH-Px: glutathione peroxidase; (**D**) GSH: reduced glutathione; (**E**) MDA: malondialdehyde. RT: Rutin (25 mg/kg body weight); RT-PNPs: Rutin-loaded nano-phytosomes (25 mg/kg body weight); EAC: Inoculated with Ehrlich Ascites Carcinoma (EAC) cells (0.2 mL); EAC/RT: Rutin (25 mg/kg body weight) + EAC cells (0.2 mL); EAC/RT-PNPs: Rutin-loaded nano-phytosomes (25 mg/kg body weight) + EAC cells (0.2 mL) (*n* = 15 for each group). Values are presented as mean ± SE. One-way ANOVA with Tukey’s post hoc test was applied. Within the same row, values bearing different superscript letters (a, b, c) differ significantly at *p* < 0.05.

Hepatic and renal antioxidant capacities were significantly reduced in EAC-treated mice, including those for reduced glutathione (GSH), superoxide dismutase (SOD), catalase (CAT), and glutathione peroxidase (GSH-Px). However, these levels were significantly improved following treatment with either crude RT or RT-PNPs, with the EAC/RT-PNP group showing greater recovery in SOD, CAT, and GSH-Px levels compared to the EAC/RT group (Figure 6 and Figure 7A–C). For GSH, no significant differences were observed between the EAC/RT and EAC/RT-PNP groups and the control groups in terms of hepatic tissue (*p* > 0.05). However, in renal tissue, GSH levels were significantly higher in the EAC/RT-PNP group than in the EAC/RT group (Figure 6 and Figure 7D). In contrast, lipid peroxidation, as indicated by malondialdehyde (MDA) levels, was significantly elevated in the EAC group (Figure 6 and Figure 7E). This elevation was markedly reduced following treatment with either form of RT, reaching the lowest levels in the EAC/RT-PNP group. No significant differences in hepatic MDA levels were observed between the EAC/RT-PNP and EAC/RT-PNP groups. However, in renal tissue, MDA levels were significantly lower in the EAC/RT-PNP group compared to the EAC/RT group. Overall, hepatic tissue exhibited higher levels of antioxidant enzymes and oxidative stress markers compared to renal tissue.

### 2.7. Inflammatory Reaction

**Figure 8:**
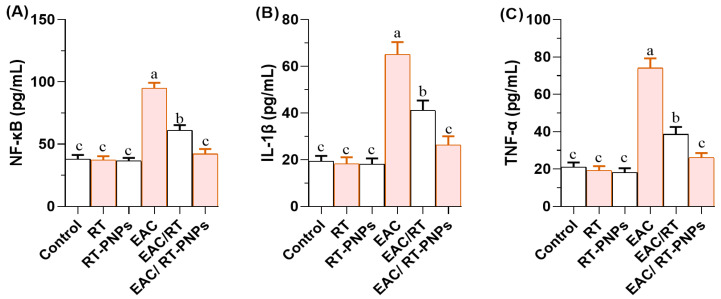
Changes in inflammatory cytokines in Ehrlich Ascites Carcinoma (EAC)-bearing mice treated with Rutin-loaded nano-phytosome and raw Rutin. (**A**) NF-κB: nuclear factor kappa B; (**B**) IL-1β: interleukin-1 beta; (**C**) TNF-α: tumor necrosis factor-alpha. RT: Rutin (25 mg/kg body weight); RT-PNPs: Rutin-loaded nano-phytosomes (25 mg/kg body weight); EAC: Inoculated with Ehrlich Ascites Carcinoma (EAC) cells (0.2 mL); EAC/RT: Rutin (25 mg/kg body weight) + EAC cells (0.2 mL); EAC/RT-PNPs: Rutin-loaded nano-phytosomes (25 mg/kg body weight) + EAC cells (0.2 mL) (*n* = 15 for each group). Values are presented as mean ± SE. One-way ANOVA with Tukey’s post hoc test was applied. Within the same row, values bearing different superscript letters (a, b, c) differ significantly at *p* < 0.05.

The levels of nuclear factor kappa B (NF-κB; Figure 8A), interleukin-1 beta (IL-1β; Figure 8B), and tumor necrosis factor-alpha (TNF-α; Figure 8C) were significantly elevated in EAC-treated mice compared to all control groups. Treatment with either crude RT or RT-PNPs significantly reduced the levels of these proinflammatory cytokines, with the most substantial reduction observed in the EAC/RT-PNP-treated group, as illustrated in Figure 8A–C. The EAC/RT-PNP group exhibited values that were not significantly different from those of the control group across all measured parameters (*p* > 0.05).

### 2.8. Expression Profiles of TP53, Bax, and Bcl-2 Genes

**Figure 9:**
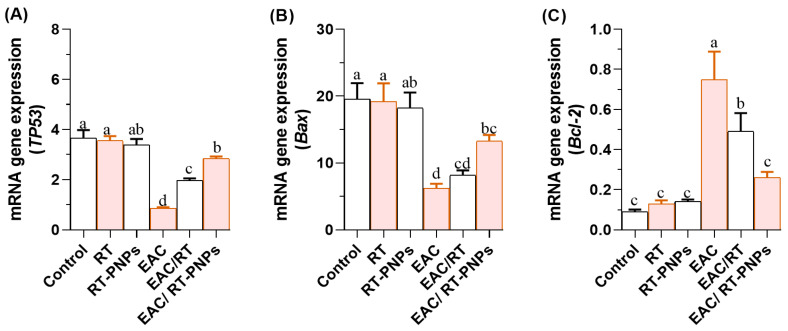
Changes in expression profiles of (**A**) *TP53*, (**B**) *Bax*, and (**C**) *Bcl-2* genes in Ehrlich Ascites Carcinoma (EAC)-bearing mice treated with Rutin-loaded nano-phytosome and raw Rutin. TP53: Tumor Protein 53; Bax: Bcl-2-associated X protein; Bcl-2: B-cell lymphoma-2. RT: Rutin (25 mg/kg body weight); RT-PNPs: Rutin-loaded nano-phytosomes (25 mg/kg body weight); EAC: Inoculated with Ehrlich Ascites Carcinoma (EAC) cells (0.2 mL); EAC/RT: Rutin (25 mg/kg body weight) + EAC cells (0.2 mL); EAC/RT-PNPs: Rutin-loaded nano-phytosomes (25 mg/kg body weight) + EAC cells (0.2 mL) (*n* = 15 for each group). Values are presented as mean ± SE. One-way ANOVA with Tukey’s post hoc test was applied. Within the same row, values bearing different superscript letters (a, b, c, d) differ significantly at *p* < 0.05.

The expression levels of the *p53*, *Bcl-2*, and *Bax* genes are presented in Figure 9A–C. The pro-apoptotic genes *Bax* and *TP53* were significantly downregulated in EAC-bearing mice. However, treatment with either crude RT or RT-PNPs significantly upregulated their expression. Notably, the *TP53* expression level in the EAC/RT-PNP group was considerably higher than that in the EAC/RT group, and it showed no significant difference compared to the negative control group. In contrast, no significant difference was observed between the EAC/RT and EAC/RT-PNP groups in terms of *Bax* expression. Concerning the anti-apoptotic *Bcl-2* gene, its expression was significantly elevated in EAC-treated mice. This upregulation was markedly reduced following treatment, with RT-PNPs producing a more pronounced downregulation than crude RT.

### 2.9. Flow Cytometric Analysis of p53 and Ki-67 Expression Results

**Figure 10:**
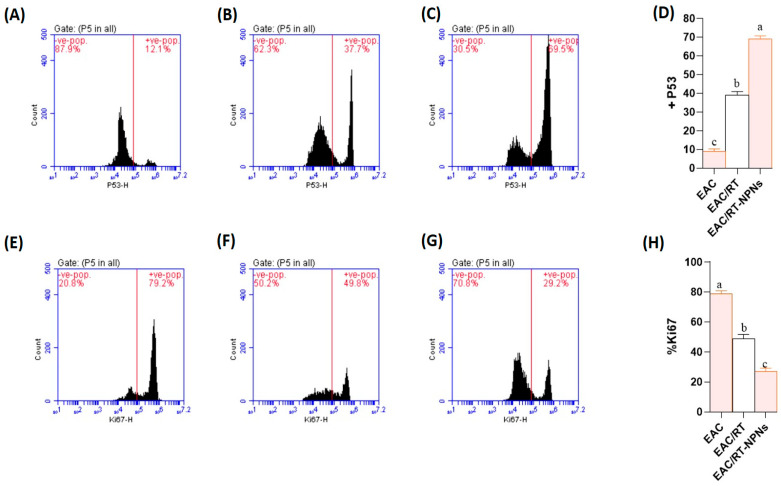
Flow cytometric analysis of the expression levels of p53 (**A**–**D**) and Ki-67 (**E**–**H**) among the experimental groups (EAC: Inoculated with Ehrlich Ascites Carcinoma (EAC) cells; EAC/RT: treated with Rutin (25 mg/kg body weight); EAC/RT-PNPs: treated with Rutin-loaded nano-phytosomes (25 mg/kg body weight), respectively, (*n* = 15 for each group). One-way ANOVA with Tukey’s post hoc test was applied. Within the same row, values bearing different superscript letters (a, b, c) differ significantly at *p* < 0.05.

Flow cytometric analysis indicated significant variations in the expression levels of p53 and Ki-67 among the experimental groups. The EAC + RT-PNP group exhibited a significantly higher percentage of p53-positive tumor cells (mean ± SD: 69.0 ± 3.4%) compared to the EAC + RT group (39.0 ± 3.9%) and the untreated EAC group (9.0 ± 2.6%) (*p* < 0.001) (Figure 10A–D). Conversely, therapy significantly reduced the expression of Ki-67, a marker of cell proliferation. The EAC + RT-PNP group exhibited the lowest Ki-67 positivity at 27.3 ± 3.3%, in contrast to the EAC + RT group at 48.8 ± 5.6% and the EAC control at 78.8 ± 3.8% (*p* < 0.001). The results indicate that RT-PNP therapy enhances p53-mediated apoptotic signaling and inhibits tumor cell growth more effectively than free RT (Figure 10E–H).

### 2.10. Histopathological Results

**Figure 11:**
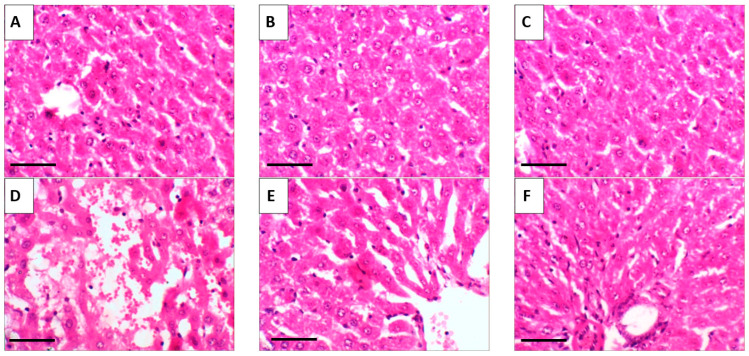
Representative photomicrographs of liver tissue from the control and experimental groups. (**A**) Control group; (**B**) RT: Rutin-treated group (25 mg/kg body weight); (**C**) RT-PNPs: Rutin-loaded nano-phytosomes (25 mg/kg body weight); (**D**) EAC: Group inoculated with Ehrlich Ascites Carcinoma (EAC) cells (0.2 mL); (**E**) EAC/RT: EAC-inoculated group treated with Rutin (25 mg/kg body weight); (**F**) EAC/RT-PNPs: EAC-inoculated group treated with Rutin-loaded nano-phytosomes (25 mg/kg body weight). All images captured at 400× magnification; scale bar = 50 µm.

**Figure 12:**
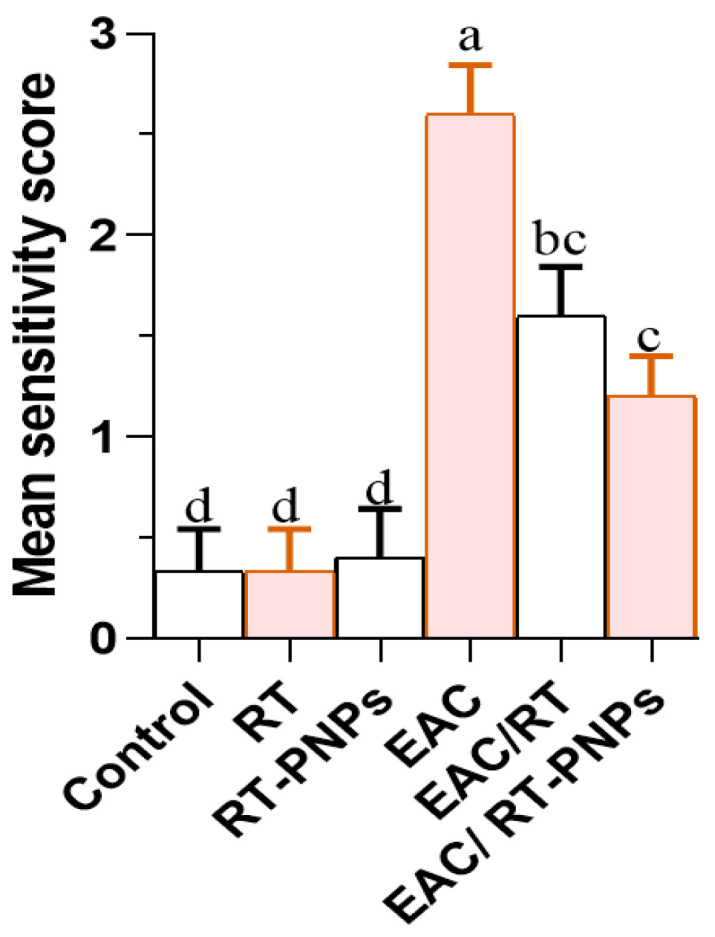
Severity scores of liver tissue damage in Ehrlich Ascites Carcinoma–bearing mice treated with raw Rutin and Rutin-loaded nano-phytosomes (RT-PNPs). Rutin (25 mg/kg body weight); RT-PNPs: Rutin-loaded nano-phytosomes (25 mg/kg body weight); EAC: Inoculated with Ehrlich Ascites Carcinoma (EAC) cells (0.2 mL); EAC/RT: Rutin (25 mg/kg body weight) + EAC cells (0.2 mL); EAC/RT-PNPs: Rutin-loaded nano-phytosomes (25 mg/kg body weight) + EAC cells (0.2 mL) (*n* = 5 for each group). Values are presented as mean ± SE. One-way ANOVA with Tukey’s post hoc test was applied. Within the same row, values bearing different superscript letters (a, b, c, d) differ significantly at *p* < 0.05.

The liver histology presented in Figure 11A–C reveals that treatment with either crude Rutin (RT) or rutin-loaded nano-phytosomes (RT-PNPs) resulted in no evident tissue damage, with liver sections appearing normal across all treated and control groups. The liver sections showed a typical hepatic structure with preserved lobular organization, intact central lobules, and hepatocytes displaying polygonal or hexagonal outlines, central nuclei, and bright eosinophilic cytoplasm. In stark contrast, the hepatic tissue of mice treated with EAC exhibited substantial histological damage, characterized by nuclear necrosis, central vein congestion, and hepatocellular vacuolar degeneration (Figure 11D). Notably, hepatic tissues from the EAC/RT and EAC/RT-PNP groups exhibited restored architecture, with well-organized lobules and hepatic cords surrounding central veins (Figure 11E,F). Figure 12 shows a notable rise in liver damage severity in the EAC group compared to the control, RT, and RT-PNP groups. Conversely, co-treatment with Rutin or RT-PNPs significantly reduced severity, with the EAC/RT-PNP group demonstrating the most pronounced improvement.

**Figure 13:**
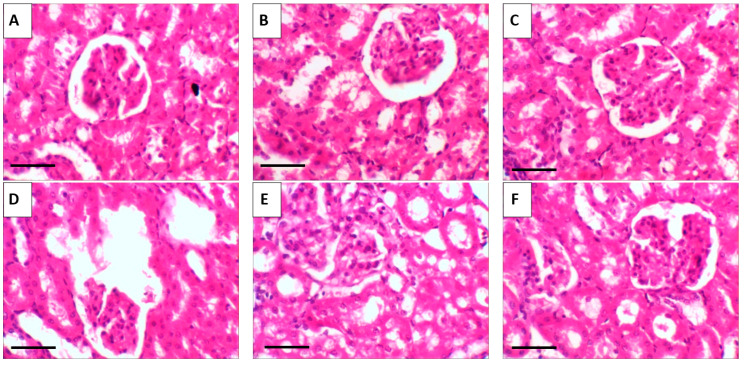
Representative photomicrographs of kidney tissue from the control and experimental groups. (**A**) Control group; (**B**) RT: Rutin-treated group (25 mg/kg body weight); (**C**) RT-PNPs: Rutin-loaded nano-phytosomes (25 mg/kg body weight); (**D**) EAC: Group inoculated with Ehrlich Ascites Carcinoma (EAC) cells (0.2 mL); (**E**) EAC/RT: EAC-inoculated group treated with Rutin (25 mg/kg body weight); (**F**) EAC/RT-PNPs: EAC-inoculated group treated with Rutin-loaded nano-phytosomes (25 mg/kg body weight). All images captured at 400× magnification; scale bar = 50 µm.

The histological examination of kidneys from control and RT- and RT-PNP-treated mice revealed typical renal morphology, showing well-structured proximal and distal tubules and healthy glomeruli (Figure 13A–C). Conversely, the EAC-treated mice demonstrated significant renal damage, characterized by periglomerular lymphocytic aggregates, congested blood vessels, and shrunken glomeruli (Figure 13D). The renal tissues of the mice in the EAC/RT and EAC/RT-PNP groups largely retained normal morphology, showing minor congestion in tubular capillaries, along with healthy glomeruli and tubules (Figure 13E,F).

**Figure 14:**
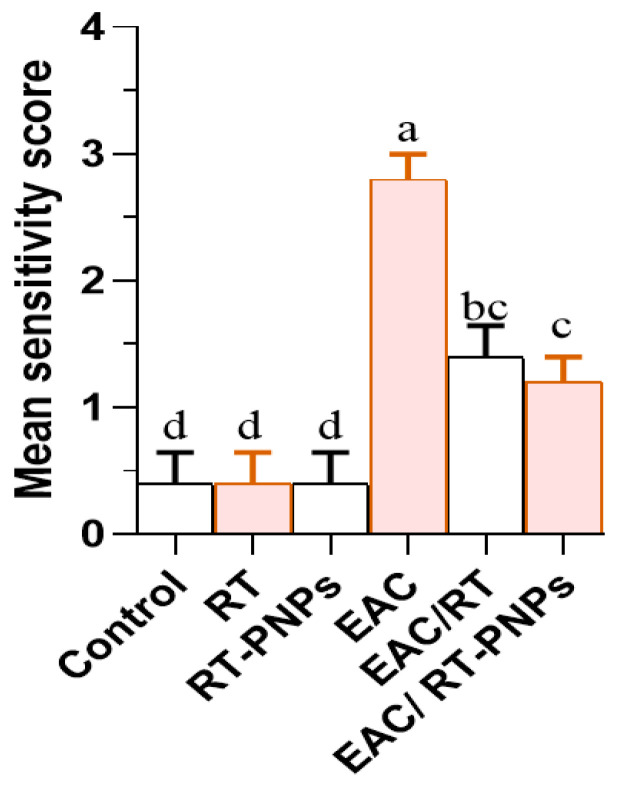
Severity scores of Kidney tissue damage in Ehrlich Ascites Carcinoma–bearing mice treated with raw Rutin and Rutin-loaded nano-phytosomes (RT-PNPs). RT: Rutin (10 mg/kg body weight); RT-PNPs: Rutin-loaded PLGA nanoparticles (10 mg/kg body weight); EAC: Inoculated with Ehrlich Ascites Carcinoma (EAC) cells (0.2 mL); EAC/RT: Rutin (10 mg/kg body weight) + EAC cells (0.2 mL); EAC/RT-PNPs: Rutin-loaded PLGA nanoparticles (10 mg/kg body weight) + EAC cells (0.2 mL) (*n* = 5 for each group). Values are presented as mean ± SE. One-way ANOVA with Tukey’s post hoc test was applied. Within the same row, values bearing different superscript letters (a, b, c, d) differ significantly at *p* < 0.05.

A significant increase in the severity of kidney damage scores was observed in the EAC group compared to the other antioxidant-treated groups. Notably, this was significantly reversed in the EAC/RT group and more effectively in the EAC/RT-PNP-treated group, thus highlighting the protective role of RT-PNPs (Figure 14).

### 2.11. Multivariable Data Analyses

**Figure 15:**
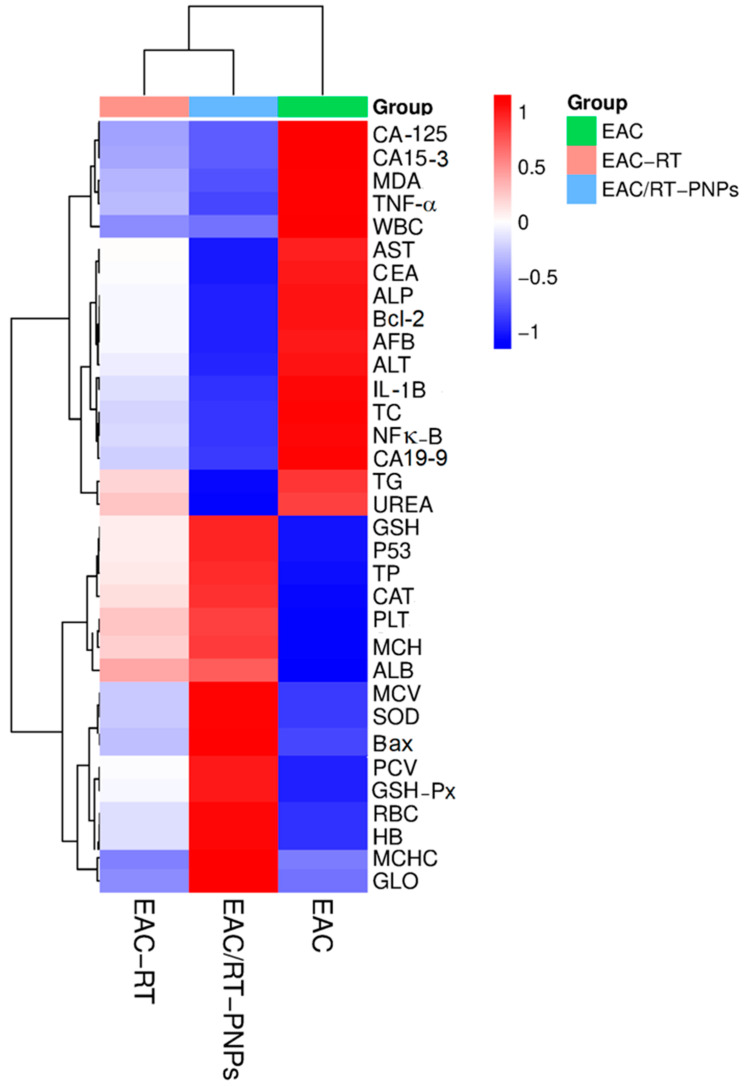
Heatmap representing hierarchical clustering of biochemical, oxidative stress, and molecular markers across EAC, EAC-RT, and EAC/RT-PNPs groups. Color gradients indicate relative expression levels (red = upregulation, blue = downregulation; Z-score normalized). The EAC group exhibited elevated tumor markers (CA19, CA125, CA15-3), inflammatory mediators (TNF, IL-1β, NF-κB), and oxidative stress indicators (MDA). In contrast, these markers were markedly reduced in EAC-RT and further suppressed in EAC/RT-PNPs. Conversely, antioxidant enzymes (SOD, GSH-Px, CAT) and apoptotic markers (P53, Bax) were notably upregulated in EAC/RT-PNPs, alongside a decline in anti-apoptotic *Bcl-2*, reflecting improved redox balance and induction of apoptosis with combined treatment.

Based on the measured biochemical and molecular parameters, hierarchical clustering and heatmap visualization indicated distinct differences among the experimental groups (EAC, EAC-RT, and EAC/RT-PNP). The bright red color indicates that the EAC group exhibited elevated levels of inflammatory markers (TNF, IL-1β, NF-κB), tumor markers (CA19, CA125, CA15), and oxidative stress indicators (MDA). Conversely, EAC-RT and, notably, EAC/RT-PNPs exhibited a notable reduction in these markers (blue gradient), indicating that the treatment inhibited tumor growth and reduced oxidative stress. Conversely, antioxidant enzymes such as SOD, GSH-Px, and CAT exhibited upregulation in the EAC/RT-PNP group, thereby indicating enhanced bodily defenses against free radicals. The EAC/RT-PNP group showed elevated levels of apoptotic markers (P53, Bax) compared to the EAC group, alongside markedly reduced levels of the anti-apoptotic marker Bcl-2. This indicates that apoptosis was activated in response to the combined treatment. The clustering patterns visually illustrate the extent to which PNPs combined with RT therapy can alter tumor biomarkers, oxidative stress, and apoptotic signaling pathways (Figure 15).

**Figure 16:**
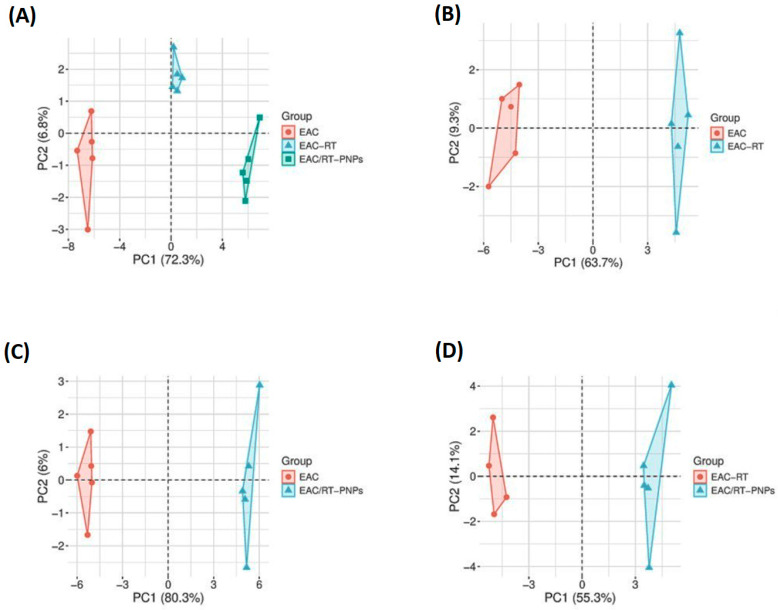
Principal component analysis (PCA) score plots showing group separation based on biomarker profiles. (**A**) EAC, EAC-RT, and EAC/RT-PNPs exhibit distinct clustering, with PC1 explaining 72.3% of the variance. (**B**) Comparison of EAC and EAC-RT (PC1 = 63.7%), (**C**) EAC and EAC/RT-PNPs (PC1 = 80.3%), and (**D**) EAC-RT and EAC/RT-PNPs (PC1 = 55.3%) confirm the clear visual clustering observed with nanoparticle-based therapy.

Distinct biomarker sets were analyzed using principal component analysis (PCA) to assess variance and grouping within the experimental groups. The initial two principal components, PC1 and PC2, accounted for 72.3% and 6.8% of the total variation in panel A, respectively, and were visually clustered into three groups (EAC, EAC-RT, and EAC/RT-PNP), indicating apparent differences in global profiles. Panel B compared EAC and EAC-RT, accounting for 63.7% of the variation in PC1, thus indicating a molecular alteration following RT treatment. Panel C indicated that PC1 accounted for 80.3% of the variance between EAC and EAC/RT-PNPs, thereby demonstrating that nanoparticle-based therapy can potentially modify tumor characteristics. PC1 (55.3%) and PC2 (14.1%) showed distinct group segregation in Panel D when comparing EAC-RT and EAC/RT-PNPs, thus indicating an enhanced effect of PNPs beyond RT treatment (Figure 16).

## 3. Discussion

The undifferentiated nature and aggressive growth of EAC cells make them particularly susceptible to chemotherapeutic agents, and they are frequently utilized in preclinical research to investigate the cytotoxic potential of new anti-cancer therapies [19]. Among natural compounds, Rutin (RT; quercetin-3-rutinoside) has garnered attention for its antioxidant and cytotoxic properties, effectively inducing cell death in EAC and other tumor cells [6]. RT’s limited water solubility presents a significant barrier to its practical application in pharmaceuticals. However, advances in drug delivery systems, particularly nanotechnology, offer promising solutions to enhance the solubility of such poorly water-soluble compounds [20]. Nanocarriers provide distinct advantages, including enhanced bioavailability, improved drug stability, and targeted delivery [21]. Phytosomes, in particular, are effective in delivering poorly water-soluble compounds through lipid integration [22]. This study focused on the formulation of RT-PNPs and the evaluation of their anti-cancer potential against EAC in comparison to crude RT, with particular emphasis on their antioxidant, pro-apoptotic, and anti-inflammatory effects. RT-PNPs demonstrated superior cytotoxic activity, significantly reducing cell viability, tumor cell count, and tumor volume while improving survival outcomes, thus showing greater efficacy than crude RT. The observed high mortality and low survival rates in EAC-bearing mice likely reflect tumor progression driven by inflammation. This inflammatory response may either enhance capillary permeability or hinder vascular and lymphatic drainage, thereby leading to the accumulation of protein-rich fluid in the peritoneal cavity [23]. The potent anti-tumor effects observed with RT-PNPs in this study may result from the dual mechanisms of inhibiting tumor cell proliferation and inducing apoptosis. Apoptosis, which is a form of programmed cell death, involves energy-dependent biochemical processes and distinct morphological changes and is essential for normal physiological functions, including immune system development, tissue remodeling, and the elimination of unwanted or damaged cells. The efficacy of numerous therapeutic agents is closely linked to their ability to influence cellular survival and apoptotic pathways [24]. In this study, RT-PNPs promoted apoptosis by downregulating *Bcl-2*, which is a gene known for its anti-apoptotic function, and upregulating the tumor suppressor genes *Bax* and *P53*. The activation of Bax and p53 plays a pivotal role in the initiation of apoptosis [25]. RT has been shown to inhibit cancer cell proliferation by triggering apoptotic pathways and inducing G2/M phase cell cycle arrest [26]. However, Rahmani et al. reported opposing effects, in which RT reduced apoptosis by downregulating apoptotic markers, including HMGB1; hsp70; caspase-3, -7, and -9; and p53, and by inhibiting oxidative stress while upregulating the anti-apoptotic protein Bcl-2 [27]. This discrepancy may be attributed to a disruption in cellular metabolism in EAC-bearing mice, which modulates the cellular response to RT, thus enhancing its pro-apoptotic role while diminishing its antioxidant action.

Tumor markers are biomolecules associated with cancer that can be detected in tissues or blood, thereby offering a non-invasive method for cancer diagnosis and monitoring. Commonly used markers, such as AFP, CEA, CA19-9, CA-125, and CA15-3, are frequently utilized in clinical oncology [28]. In the present study, mice bearing EAC tumors exhibited markedly increased levels of these markers, likely reflecting the aggressive and metastatic nature of EAC cells, which can trigger the formation of a range of abdominal malignancies, including those of the liver, stomach, pancreas, colon, and ovaries [29]. In contrast, RT-PNP-treated mice showed a notable decline in tumor marker levels, which may be due to RT’s ability to suppress tumor growth, induce apoptosis, block new blood vessel formation, reduce inflammation, and strengthen antioxidant defense mechanisms [26]. The enhanced effect observed with RT-PNPs compared to crude RT highlights the benefits of nanoparticle formulation, including improved bioavailability, absorption, and stability.

This study demonstrated that EAC-bearing mice exhibited macrocytic normochromic anemia, which is characterized by reduced RBC/platelet counts and hemoglobin levels. These findings are consistent with those of Elsadek et al. [30], suggesting that EAC may impair erythropoiesis due to folate or iron deficiency, which is potentially aggravated by the rapid consumption of folic acid by proliferating tumor cells for DNA/RNA synthesis and thiamine depletion [31]. The thrombocytopenia observed in this study may be explained by EAC-driven bone marrow suppression [32]. Additionally, the elevated WBC count in EAC-bearing mice is likely a consequence of tumor-related oxidative stress and inflammation, with reactive oxygen species playing a pivotal role in DNA damage and the onset of cancer [33]. This immune alteration may be partially attributed to the overexpression of tumor necrosis factor-alpha, which disrupts leukocyte adhesion and migration, impairs macrophage function, and interferes with lymphocyte maturation and hematopoiesis, which are factors that may explain both anemia and leukocytosis [34]. The administration of RT-PNPs resulted in significant improvements in hematological indices, restoring hemoglobin levels, RBC counts, and WBC counts to levels comparable to those observed in healthy control mice. These results suggest a potential hematoprotective effect of RT-PNPs, which is likely due to their ability to counteract inflammation and oxidative damage [15]. Similarly, Han et al. reported that RT promotes hematopoietic recovery by reducing stem cell apoptosis, limiting oxidative stress, and fostering tissue regeneration and proper differentiation [35].

This study demonstrated a gradual increase in body weight accompanied by cancer progression, primarily due to the excessive accumulation of ascitic fluid. This accumulation is attributed to an inflammatory response, along with tissue fibrosis related to collagen deposition [36]. Such pathological changes severely impair hepatocyte function and compromise hepatic integrity in EAC-bearing mice. Supporting this, the activities of liver enzymes, including AST, ALT, and ALP, were significantly elevated, thus indicating hepatic dysfunction. Additionally, EAC tumors appear to promote the dissemination of pro-angiogenic factors and stimulate peritoneal angiogenesis, thereby exacerbating fibrosis and hepatic inflammation [37]. The elevated levels of TG and TC observed in EAC-bearing mice likely indicate enhanced lipogenesis and disrupted lipid homeostasis, which are processes commonly associated with tumor development [38]. Notably, treatment with RT-PNPs effectively regulated lipid metabolism, as indicated by marked decreases in TC and TG levels. Additionally, RT-PNP administration resulted in substantial improvements in liver function, as evidenced by significant reductions in AST, ALT, and ALP levels in blood serum, thus outperforming the effects of crude RT. Decreased serum albumin and globulin levels in EAC-bearing mice suggest impaired hepatic protein synthesis resulting from tumor-induced liver injury [39]. Histopathological analysis corroborated this, revealing marked hepatic degeneration in untreated mice. Remarkably, treatment with RT-PNPs preserved normal liver morphology and ultrastructure, which is characterized by organized hepatic cords and well-aligned hepatocytes, likely due to the antioxidant-mediated protection of hepatic cells and enhancement in their synthetic function. EAC-induced oxidative stress was evident in the liver tissue of affected mice, as indicated by elevated MDA levels, a substantial decrease in GSH content, and the reduced activity of key antioxidant enzymes, including SOD, GSH-Px, and CAT. The increased MDA levels reflect enhanced lipid peroxidation, which is likely due to the accumulation of thiobarbituric acid-reactive substances and/or excessive production of ROS, both of which contribute to cellular macromolecule damage [40]. Increased lipid peroxidation in EAC-bearing mice has been associated with elevated cholesterol levels [39]. The observed reductions in SOD and GSH-Px activities may result from a decreased erythrocyte GSH content, which compromises immune function and increases cancer risk [41]. Moreover, diminished SOD activity may be linked to the loss of mitochondrial Mn-SOD in tumor cells, thereby contributing to further hepatic antioxidant exhaustion [42]. RT-PNP administration effectively countered oxidative damage by increasing GSH-Px, CAT, and SOD activities; restoring GSH content; and reducing MDA levels in hepatic tissue, which is in agreement with the findings of Suraweera et al. [43], who found that RT mitigates oxidative stress through the Nrf2/ARE pathway and enhances antioxidant enzyme activity, and with those of Emeka et al., who observed that RT reduces ROS and lipid peroxidation [44].

Concerning kidney function, the present findings indicate substantial renal dysfunction in EAC-bearing mice, as evidenced by elevated serum levels of urea and creatinine. These biochemical alterations were corroborated by histopathological analysis, which revealed degenerative, inflammatory, and vascular abnormalities, in addition to neoplastic cell infiltration in renal tissues. These results are consistent with those shown by Donia et al. [45] and Abd Eldaim et al. [46], who similarly reported EAC-associated kidney damage. These alterations likely result from tumor metastasis-related renal injury, which disrupts tubular reabsorption and glomerular filtration, thereby reducing the clearance of urea and creatinine and leading to their accumulation in the bloodstream. Moreover, these changes are further exacerbated by oxidative stress, which serves as a key driver of this renal pathology observed in EAC models [47]. Oxidative stress is widely acknowledged as a key contributor to both EAC-induced renal injury and cancer progression [48]. The overproduction of ROS, inflammatory mediators, and tumor-induced catabolic activity accelerates renal damage. This effect is further intensified by the kidneys’ high content of long-chain polyunsaturated fatty acids, which makes them highly susceptible to oxidative lipid damage [49]. Our results are supported by those of Medhat et al. [50] and Donia et al. [45], who observed increased renal MDA levels along with significant reductions in GSH content and the activities of SOD, GSH-Px, and CAT in EAC-bearing mice, underscoring oxidative stress-induced renal impairment. Chronic ROS generation not only enhances lipid peroxidation but also suppresses intrinsic antioxidant defenses, thereby contributing to progressive renal injury and facilitating tumor progression [32].

RT-PNP administration in EAC-bearing mice led to notable reductions in serum urea and creatinine, enhanced activity of key antioxidant enzymes (SOD, CAT, and GSH-Px), and suppressed lipid peroxidation, as evidenced by decreased MDA levels, indicating renal protection. This effect was more pronounced than that observed with crude RT, likely due to its superior ROS-scavenging activity in nanoparticle form. These improvements were reinforced by the microscopic and ultrastructural findings, which primarily showed intact renal tubules and glomeruli, with only minor congestion of the tubular vessels. In comparison, untreated EAC-bearing mice exhibited significant structural damage, as evidenced by periglomerular lymphocyte infiltration and severe blood vessel congestion.

There is a well-established association between chronic inflammation and the development of malignancies across various anatomical sites. Evidence suggests that proinflammatory pathways contribute to cancer initiation and progression, with the levels of biomarkers like NF-κB and proinflammatory cytokines commonly being elevated in chronic inflammatory environments, thereby contributing to carcinogenic transformation [15]. The interplay of inflammatory pathways generates a microenvironment that promotes the uncontrolled proliferation of cancer cells [37]. This facilitates both the genetic mutations necessary for cancer development and the conditions that enable tumor growth [51]. Among the primary molecular regulators, NF-κB signaling has been identified as a central regulator of inflammatory responses, orchestrating the expression of numerous proinflammatory cytokines such as IL-1β and TNF-α, as well as a broad spectrum of inflammatory mediators [52]. Similarly, the present results demonstrated a significant increase in inflammatory markers in EAC-injected mice, as evidenced by the elevated serum levels of NF-κB, IL-1β, and TNF-α compared to healthy controls. However, the administration of RT-PNPs notably attenuated these inflammatory markers, which may stem from RT’s antioxidant capacity and its ability to interfere with key inflammatory signaling cascades, including the inhibition of NF-κB activation. Consistent with our findings, Muvhulawa et al. reported that RT suppresses inflammation by inhibiting NF-κB and MAPK signaling pathways and by reducing the levels of IL-1β, IL-6, TNF-α, and COX-2 [15].

Multivariate analyses, including hierarchical clustering and PCA, demonstrate the systemic effects of various treatment modalities. The EAC group exhibited elevated levels of inflammatory cytokines, tumor markers, and indicators of oxidative stress. In contrast, EAC/RT-PNPs demonstrated a significant suppression of these markers, along with enhanced antioxidant defense and induction of apoptosis. PCA demonstrated visually discriminated groups along principal components, thus reflecting observable treatment-induced variance. The most important shift occurred between EAC and EAC/RT-PNPs, thereby highlighting the enhanced effect of nanoparticle-based therapy combined with RT on tumor biology and homeostatic equilibrium. The separation of EAC-RT and EAC/RT-PNPs highlights the advantages of nanoparticle integration beyond the effects of RT, thereby supporting biochemical and molecular advantages.

This study has some limitations that should be acknowledged. First, stability under prolonged storage conditions was not evaluated, which is essential for practical applications. Second, a blank phytosome/empty nanoparticle control was absent, which would have provided greater clarity on the independent contribution of the carrier system. Third, the results are derived from a single animal tumor model, which restricts the generalizability of the findings. Therefore, the translational potential of RT and its nanoparticle formulation should be interpreted with caution, and further validation in clinical settings and additional models will be essential to confirm these preliminary observations.

Additionally, only female mice were utilized to reflect the mammary (breast) cancer origin of the EAC model and its greater clinical relevance to human breast cancer, which predominantly affects females. However, considering emerging evidence for sex-based differences in cancer biology, progression, and response to therapy, future investigations using both male and female models are recommended to characterize sex-specific effects fully and to improve the generalizability of the preclinical findings.

## 4. Materials and Methods

### 4.1. Preparation of Rutin-Loaded Phytosome Nanoparticles (RT-PNPs)

The thin-layer hydration technique was employed to formulate RT-PNPs. The formulation consisted of RT, cholesterol, and phosphatidylcholine in optimized ratios. Specifically, cholesterol was incorporated at 5–10% (*w*/*w*) of the total lipid content, while RT (25 mg/kg equivalent) was added at a molar ratio of 1:2 with phosphatidylcholine. Phosphatidylcholine was chosen as the primary carrier due to its biocompatible and amphiphilic features, which enhance intestinal absorption and facilitate stable phytosome formation. Cholesterol was included to strengthen and reduce drug leakage, membrane rigidity, and prolong colloidal stability. In contrast, RT was the active flavonoid of interest, selected for its potent anti-cancer and antioxidant properties.

For preparation, phosphatidylcholine and RT were dissolved in methanol, while cholesterol was dissolved in dichloromethane. The solutions were mixed in a round-bottom flask and subjected to rotary evaporation (Heidolph, Schwabach, Germany) at 45 °C and 100 rpm for approximately 1 h, until the complete elimination of organic solvents resulted in a thin lipid film. Residual solvents were removed via vacuum drying, and the film was flushed with nitrogen before being left overnight at 25 °C. The dried film was then hydrated with distilled water in a rotary evaporator at 45 °C. Particle size reduction was achieved via homogenization at 20,000 rpm (Heidolph, Schwabach, Germany), followed by probe sonication (Sonix, Vibracell, Newtown, CT, USA).

A range of formulations was designed to optimize the RT-PNPs by adjusting the cholesterol level (0%, 5%, and 10% *w*/*w* of total lipid) and altering the PC: RT molar ratio (1:1, 1:2, 1:3). The prepared formulations (F1–F7) underwent evaluation for key critical quality attributes (CQAs), focusing on particle size, zeta potential, polydispersity index (PDI), and encapsulation efficiency (EE, %). The formulation demonstrating the lowest particle size and PDI, together with the highest EE% and absolute zeta potential, was chosen as the optimized RT-PNPs.

The CQAs of the prepared RT-PNPs included particle size, zeta potential, polydispersity index (PDI), and entrapment efficiency, as these parameters are essential indicators of nanoparticle reproducibility, stability, and functional performance. To assess internal morphology, freshly prepared RT-PNPs were imaged using a TEM (JEOL JEM-2100, JOEL, Tokyo, Japan) at an accelerating voltage of 200 kV. Digital Micrograph and Soft Imaging System Viewer software, Gatan Microscopy Suite (DigitalMicrograph) version 3.62 (Gatan, Inc., Pleasanton, CA, USA), were used for image analysis.

To verify the compatibility and rule out possible chemical interactions between RT and the carrier components, Fourier Transform Infrared Spectroscopy (FTIR; Bruker Tensor 27, Bremen, Germany) was employed. Spectral data were collected across 4000–400 cm^−1^, and the characteristic absorption peaks of RT were compared with those of the unloaded carrier system and the formulated RT-PNPs. The absence of significant peak shifts indicated the successful encapsulation of RT without chemical incompatibility.

Particle size and zeta potential were measured using dynamic light scattering (DLS) on a Zetasizer Nano ZS (Malvern Instruments, Malvern, UK). Measurements were performed at 25 °C following suitable dilution with deionized water.

Encapsulation efficiency (EE%) was assessed using ultracentrifugation at 20,000 rpm for 30 min at 4 °C to separate the unentrapped RT. The amount of free RT in the supernatant was quantified spectrophotometrically at 257 nm, and EE% was calculated using the following equation:

EE% = [(Total RT − Free RT)/Total RT] × 100

The optimized formulation was chosen based on its narrow polydispersity index (PDI < 0.3), the lowest particle size, the highest absolute zeta potential (|>25| mV), and the highest encapsulation efficiency, which collectively capture the stability and robustness of the nanosystem. Fourier-transform infrared (FTIR) spectroscopy was employed to assess potential interactions and verify the compatibility of RT with the excipients. Spectra were collected for crude RT, cholesterol, phosphatidylcholine, their physical mixture, and the optimized RT-PNPs using a Bruker Tensor 27 spectrophotometer. Sample preparation involved the KBr pellet method, with data acquired between 4000 and 400 cm^−1^ at a resolution of 4 cm^−1^.

### 4.2. Induction of EAC in Mice

The Ehrlich ascites carcinoma (EAC) cell line was obtained from the National Cancer Institute (NCI), Cairo, Egypt, and routinely sustained in vivo through the serial intraperitoneal transplantation of 2.5 × 10^6^ cells into female Swiss albino mice every 10 days following established protocols [6].

Only female mice were used, as the EAC model represents murine mammary cancer and most accurately recapitulates breast cancer biology, including tumor progression and therapeutic responsiveness, in females. This aligns with the clinical epidemiology of breast cancer and with established EAC research practices, thus providing greater translational relevance for anti-tumor investigations [19,53].

A ketamine–xylazine combination (80 mg/kg and 10 mg/kg, respectively) was administered intraperitoneally to anesthetize the mice. Ascitic fluid was then obtained from EAC-inoculated mice on day 7 or 8 post-injection, using the same anesthetic regimen. Following collection, the ascitic fluid was analyzed for viable cell count using a hemocytometer in conjunction with 0.4% trypan blue dye exclusion. The cell concentration was then adjusted to 2.5 × 10^6^ viable cells per 0.2 mL of PBS for the subsequent inoculation.

### 4.3. Animal Handling and Experimental Protocol

Ninety healthy female Swiss albino mice (Mus musculus) with an average body weight of 19.2 g were supplied by the animal House, Faculty of Medicine, Mansoura University, Egypt. The animals were young adults, approximately 6–8 weeks old, at the beginning of this experiment. Animals were maintained in groups in standard plastic cages under controlled conditions: relative humidity was maintained at around 45%, the temperature was kept at 25 °C, and a 12 h light/dark cycle was used. Food and water were provided ad libitum throughout this study. Before initiating the experimental procedures, all animals were allowed a one-week acclimatization period to adapt to the laboratory conditions. All procedures were conducted following the Guide for the Care and Use of Laboratory Animals (8th edition, NRC, 2011) and the International Guiding Principles for Biomedical Research Involving Animals (1985). All animal experiments were approved by the Research Ethics Committee, Faculty of Veterinary Medicine, Mansoura University, Egypt (registration code number MU-ACUC; VM.R.25.08.242).

Six groups of 15 mice were formed through random assignment. The first group remained untreated as a control, while the second group was administered Rutin (RT) at a daily oral dose of 25 mg/kg body weight for 20 days. The third group received RT-loaded nano-phytosomes (RT-PNPs) at the same dose and duration as the free RT group, as determined by the LD_50_ reported by Yılmaz et al. [6]. The fourth group, referred to as the EAC group, was administered 0.2 mL of EAC cells (2.5 × 10^6^ cells) via intraperitoneal injection on the first day and remained untreated for the following 20 days. The fifth group (EAC + RT) was treated with Rutin daily for 20 days after EAC cell inoculation. The sixth group (EAC + RT-PNPs) received RT-PNPs daily over the same period.

The sample size for each experimental group (n = 15 mice) was determined based on previously published EAC murine studies, in which 10–12 mice per group were used to detect biological and tumor-related differences reliably. To ensure at least 80% power for pairwise comparisons at α = 0.05 and to account for anticipated attrition (10–20%, e.g., post-EAC mortality), we increased the group size to 15. This design is consistent with that needed to plan a one-way ANOVA with six groups, in which a total sample size of n = 90 yields approximately 80% power to detect moderate to large effects, which is in line with international standards for preclinical research and adheres to ethical principles that minimize animal use [54,55]. There were no specific a priori inclusion or exclusion criteria established. All animals enrolled at the beginning of the study were included in the experiment and completed it.

Body weight measurements were taken for each animal at baseline and at the end of the experiment period. The survival of EAC-bearing mice in all groups was assessed through daily observation. The mean survival time (MST) was determined using the following equation: MST = (first death + last death)/2 [23].

### 4.4. Sample Collection

Mice from each experimental group were euthanized via cervical dislocation at the end of the experimental period. To ensure consistency in blood sampling, all animals were fasted for 10 h and anesthetized with inhaled tetrahydrofuran to minimize distress. Retro-orbital venous sinus puncture was used to collect blood samples, which were then split into two portions: one in an EDTA tube for complete blood count analysis and the other in a plain tube for serum separation. The isolated serum was stored under appropriate conditions for detailed biochemical and inflammatory cytokine evaluations.

### 4.5. Hematological Profile Analysis

Hematological analysis was conducted using the Hema Screen 18 automated analyzer (Hospitex Diagnostics, Tuscany, Italy) to measure key indices, including red blood cell (RBC) count, hemoglobin (Hb) level, packed cell volume (PCV), platelet count (PLT), total white blood cell (WBC) count, mean corpuscular hemoglobin (MCH), mean corpuscular volume (MCV), and mean corpuscular hemoglobin concentration (MCHC). In addition, blood cells were manually counted using an improved Neubauer hemocytometer. Hayem’s solution was employed for erythrocyte analysis, while Turk’s solution was used to stain leukocytes. Reference normal ranges for female Swiss albino mice (BW: 29.4 ± 1.2 g; RBC: 7.74 ± 0.69 × 10^6^/µL; HCT: 40.7 ± 1.7%; HGB: 13.45 ± 0.71 g/dL; MCV: 54.6 ± 5.2 fL; MCH: 17.5 ± 2.2 pg; MCHC: 32.6 ± 1.0 g/dL; RDW-CV: 16.6 ± 1.7%; RDW-SD: 31.2 ± 2.8 fL) were extracted from published laboratory references [56]. These ranges allow for a contextual interpretation of the hematological changes observed in the experimental groups.

### 4.6. Serum Biochemical Analysis

A biochemical analysis of serum samples was performed using standardized commercially available kits, following the manufacturers’ guidelines. The following parameters were quantified: total protein (TP; Cat. No. CBP007-K), albumin (Cat. No. MET-5017), triglycerides (TGs; Cat. No. TR 200), total cholesterol (TC; Cat. No. STA-384), aspartate aminotransferase (AST; Cat. No. MET-5127), alanine aminotransferase (ALT; Cat. No. MET-5123), alkaline phosphatase (ALP; Cat. No. CBA-301), urea (Cat. No. STA-382), and creatinine (STA-378). The concentration of globulin was determined by subtracting the albumin concentration from the total protein concentration. The serum concentrations of interleukin-1 beta (IL-1β), tumor necrosis factor-alpha (TNF-α), and nuclear factor kappa B (NF-κB) were measured using commercial ELISA kits (Cat. No. MBS825017, MBS267737, and MBS287521, respectively) supplied by MyBioSource, San Diego, CA, USA. All procedures were carried out strictly in accordance with the manufacturer’s instructions. Alpha-fetoprotein (AFP) levels were measured using an automated quantitative enzyme-linked fluorescent assay (ELFA) with the mini-VIDAS^®^ AFP system (bioMérieux, Marcy-l’Étoile, France). The serum levels of carcinoembryonic antigen (CEA), cancer antigen 19-9 (CA 19-9), cancer antigen 125 (CA-125), and cancer antigen 15-3 (CA 15-3) were quantified using a quantitative sandwich immunoassay with mouse-specific ELISA kits (MyBioSource, San Diego, CA, USA).

### 4.7. Quantification of Tumor Volume and Viable Cell Count in Ascitic Fluid

Following EAC inoculation, ascitic fluid was aspirated from each mouse and centrifuged at 1200 rpm for 5 min. The volume of the resulting tumor cell pellet was measured using a graduated conical centrifuge tube. Cell viability was determined via the trypan blue exclusion method, in which viable cells excluded the dye and remained unstained, whereas non-viable cells were stained blue.

### 4.8. Antioxidant Status

Following dissection, liver and kidney tissues were carefully washed with ice-cold 1.15% potassium chloride (KCl) solution to eliminate any remaining blood and tissue debris. Tissues designated for biochemical assessments were immediately frozen at −20 °C, while representative portions were fixed in 10% neutral-buffered formalin for subsequent histopathological evaluation. Liver and kidney tissues were homogenized in ice-cold phosphate buffer (pH 7.4) and centrifuged at 4000 rpm for 15 min at 4 °C to obtain clear supernatants. These were subsequently used to quantify antioxidant enzymes and oxidative stress parameters. The collected supernatants were used to determine the levels of antioxidant enzymes, including catalase (CAT, Cat. No. CA 2517), superoxide dismutase (SOD, Cat. No. SD 2521), and glutathione peroxidase (GSH-Px, Cat. No. GP 2524), as well as reduced glutathione (GSH, Cat. No. GR 2511). Malondialdehyde (MDA) levels, as a marker of lipid peroxidation, were measured using the thiobarbituric acid reactive substances (TBARS) method (MDA, Cat. No. MD 2529). All measurements were conducted using commercially available colorimetric assay kits (Biodiagnostic Co., Giza, Egypt) following the manufacturer’s instructions.

### 4.9. Expression Levels of P53, Bax, and Bcl2 Genes

Total cellular RNA was extracted from the tumor cells of mice bearing EAC using the Thermo Scientific Gene JET^TM^ RNA Purification Kit (Thermo Scientific, Waltham, MA, USA), following the manufacturer’s instructions. Five micrograms of purified RNA were reverse-transcribed into complementary DNA (cDNA) using the RevertAid First Strand cDNA Synthesis Kit (Thermo Scientific, Waltham, MA, USA), following the provided protocol. Primers for the amplification of the *P53*, *Bax*, *Bcl-2*, and *GAPDH* genes (Table 4) were designed using NCBI Primer-BLAST and synthesized via Invitrogen (Carlsbad, CA, USA). GAPDH was used as the internal control (housekeeping gene) for normalizing gene expression levels. Primers targeting the *GAPDH*, *Bax*, *Bcl-2*, and *P53* genes were adopted from the study by Mohamed et al. [57].

### 4.10. Flow Cytometric Analysis of p53 and Ki-67 Expression

Single-cell suspensions were prepared from tumor tissues by mechanically disrupting the tissues, filtering through a 70 µm mesh (Corning Inc., Corning, NY, USA, Cat# 352350), and centrifuging at 300×g for 5 min at 4 °C. We fixed and permeabilized 1 × 10^6^ cells using the BD Cytofix/Cytoperm^TM^ Kit (BD Biosciences, San Jose, CA, USA, Cat# 554714) according to the manufacturer’s instructions. Cells were stained with Alexa Fluor^®^ 488-conjugated anti-p53 (clone DO-7, Cell Signaling Technology, Danvers, MA, USA, Cat# 48818) and PE-conjugated anti-Ki-67 (clone SolA15, Thermo Fisher/eBioscience, Waltham, MA, USA, Cat# 12-5698-82) for 30 min at 4 °C in the dark. Following two washes in Perm/Wash buffer, the cells were resuspended in staining buffer and analyzed using a BD FACSCalibur^TM^ cytometer (BD Biosciences, San Jose, CA, USA), which recorded 10,000 events per sample. Data processing was conducted using FlowJo^TM^ v10 (Tree Star Inc., Ashland, OR, USA). The findings were presented as the percentage of positive cells and the mean fluorescence intensity (MFI). Isotype and fluorescence minus one (FMO) controls were employed to ensure the accuracy of the gating.

### 4.11. Histopathological Analysis

Liver and kidney tissues fixed in 10% neutral-buffered formalin were dehydrated in ascending ethanol concentrations (70%, 80%, 90%, and 100%), each for one hour. Following dehydration, the tissues were cleared in xylene (two changes, each for 1 h) and then embedded in paraffin blocks for sectioning. Tissue sections approximately 5 μm thick were prepared using a rotary microtome, stained with hematoxylin and eosin (H&E), and examined microscopically. Representative fields were photographed using a light microscope equipped with a high-resolution digital imaging system. Liver and kidney lesions were assessed in five mice using three sections per slide. Each section was examined at 400× magnification in four randomly selected fields, resulting in twelve fields per organ for semi-quantitative analysis. Histopathological lesions were scored using a standardized scale (0 = none; 1 = mild; 2 = moderate; 3 = severe), as summarized in Table 5 and Table 6. For each mouse, a mean lesion score was obtained by averaging scores from all observed fields in the liver and kidney tissues.

### 4.12. Statistical Analysis

Normal distribution and equal variance assumptions were evaluated using the Shapiro–Wilk test and Levene’s test, respectively. Differences among groups were analyzed via a one-way ANOVA using SAS version 9.3 (Proc ANOVA; SAS Institute, Cary, NC, USA, 2012). Significant ANOVA results were further analyzed using Tukey’s post hoc test to determine differences between groups. All values are reported as the mean ± standard error, with significance defined as *p* < 0.05. Data were visualized using GraphPad Prism 9.0 (GraphPad Software Inc., La Jolla, CA, USA), with multivariate statistical analyses such as heatmap clustering and principal component analysis (PCA) performed using SRplot (https://www.bioinformatics.com.cn/en) (accessed on 29 July 2025).

## 5. Conclusions

The toxicity induced by Ehrlich ascites carcinoma is associated with marked disturbances in hematological and biochemical parameters, elevated oxidative stress, diminished antioxidant defenses, dysregulated apoptotic responses, and increased levels of inflammation-related cytokines, accompanied by pronounced pathological lesions in hepatic and renal tissues. Compared with crude RT, RT-PNPs more effectively preserved hepatorenal architecture, enhanced antioxidant capacity, and normalized key biochemical and inflammatory markers. Furthermore, RT-PNPs demonstrated enhanced effect in promoting apoptosis and inhibiting tumor growth. These results highlight the therapeutic advantages of nanoformulated RT, which are attributable to its improved stability, permeability, and aqueous solubility, thereby optimizing its anti-cancer potential.

## Figures and Tables

**Table 1:** Optimization of RT-PNPs formulations. Effect of cholesterol content and phosphatidylcholine (PC): Rutin molar ratio on polydispersity index (PDI), particle size, zeta potential, and encapsulation efficiency.

Formulations	PC: RT Molar Ratio	Cholesterol (% *w*/*w* of Total Lipid)	PDI	Particle Size (nm, DLS)	Zeta Potential (mV)	Encapsulation Efficiency (EE%)
F1	1:1	0%	0.471	262	−22.3	66.31
F2	1:1	5%	0.447	231	−27.9	71.52
F3	1:1	10%	0.423	224	−29.5	74.03
F4	1:2	0%	0.401	201	−32.4	79.24
F5	1:2	5%	0.392	185	−37.0	84.62
F6	1:2	10%	0.410	193	−35.2	83.14
F7	1:3	5%	0.452	214	−31.1	78.47

**Table 2:** Comparative effects of Rutin-loaded nano-phytosomes and raw Rutin on hematological profiles in mice with Ehrlich ascites carcinoma.

Parameters	Control	RT	RT-PNPs	EAC	EAC/RT	EAC/RT-PNPs
RBCs (10^6^/μL)	7.14 ± 0.29 ^a^	7.22 ± 0.34 ^a^	7.26 ± 0.39 ^a^	4.15 ± 0.22 ^c^	5.21 ± 0.28 ^b^	6.85 ± 0.30 ^a^
Hb (g/dL)	13.8 ± 0.62 ^a^	14.02 ± 0.59 ^a^	14.21 ± 0.44 ^a^	8.84 ± 0.48 ^b^	10.23 ± 0.53 ^b^	12.36 ± 0.76 ^a^
PLT (×10^3^/µL)	534.12 ± 6.23 ^a^	539.37 ± 7.14 ^a^	540.23 ± 7.25 ^a^	336.21 ± 6.36 ^c^	465.21 ± 8.96 ^b^	520.39 ± 10.15 ^a^
PCV (%)	46.25 ± 2.33 ^ab^	47.21 ± 3.15 ^ab^	48.53 ± 4.24 ^a^	30.21 ± 2.20 ^c^	36.26 ± 3.02 ^bc^	42.58 ± 5.15 ^ab^
WBC (10^3^/μL)	8.62 ± 0.32 ^b^	8.56 ± 0.29 ^b^	8.44 ± 0.35 ^b^	14.21 ± 0.69 ^a^	9.32 ± 0.78 ^b^	9.03 ± 0.68 ^b^
MCH (pg)	19.32 ± 0.08 ^b^	19.42 ± 0.09 ^b^	19.57 ± 0.44 ^b^	21.30 ± 0.03 ^a^	19.63 ± 0.04 ^b^	18.03 ± 0.32 ^c^
MCHC (%)	34.69 ± 2.03 ^a^	35.21 ± 3.45 ^a^	36.23 ± 3.92 ^a^	31.15 ± 4.63 ^a^	31.16 ± 2.88 ^a^	34.22 ± 3.42 ^a^
MCV (fl)	55.82 ± 3.03 ^a^	55.53 ± 5.74 ^a^	54.58 ± 7.18 ^a^	69.40 ± 10.34 ^a^	63.38 ± 6.01 ^a^	52.99 ± 4.38 ^a^

RBCs: Red blood cells; Hb: Hemoglobin; PLT: Platelet count; PCV: Packed cell volume; WBCs: White blood cells; MCH: Mean corpuscular hemoglobin; MCHC: Mean corpuscular hemoglobin concentration; MCV: Mean corpuscular volume. RT: Rutin (25 mg/kg body weight); RT-PNPs: Rutin-loaded nano-phytosomes (25 mg/kg body weight); EAC: Inoculated with Ehrlich Ascites Carcinoma (EAC) cells (0.2 mL); EAC/RT: Rutin (25 mg/kg body weight) + EAC cells (0.2 mL); EAC/RT-PNPs: Rutin-loaded nano-phytosomes (25 mg/kg body weight) + EAC cells (0.2 mL). Within the same row, values bearing different superscript letters (a, b, c) differ significantly at *p* < 0.05. Values are presented as mean ± SE.

**Table 3:** Comparative effects of Rutin-loaded nano-phytosome and raw Rutin on blood chemistry in mice with Ehrlich ascites carcinoma.

Parameters	Control	RT	RT-PNPs	EAC	EAC/RT	EAC/RT-PNPs
TP (g/dL)	6.74 ± 0.31 ^a^	6.82 ± 0.29 ^a^	6.91 ± 0.32 ^a^	4.37 ± 0.19 ^c^	5.44 ± 0.21 ^b^	6.21 ± 0.28 ^ab^
Glo (g/dL)	2.77 ± 0.17 ^a^	2.81 ± 0.18 ^a^	2.72 ± 0.22 ^a^	2.09 ± 0.13 ^b^	2.13 ± 0.03 ^b^	2.67 ± 0.12 ^a^
Alb (g/dL)	3.97 ± 0.14 ^a^	4.01 ± 0.11 ^a^	4.19 ± 0.10 ^a^	2.28 ± 0.06 ^c^	3.31 ± 0.18 ^b^	3.54 ± 0.16 ^b^
TG (mg/dL)	86.44 ± 2.29 ^c^	83.32 ± 3.55 ^c^	84.57 ± 4.14 ^c^	153.32 ± 5.25 ^a^	133.62 ± 4.93 ^b^	97.41 ± 5.37 ^c^
TC (mg/dL)	118.41 ± 3.23 ^c^	121.12 ± 4.12 ^c^	123.27 ± 4.97 ^c^	183.33 ± 5.33 ^a^	144.54 ± 4.36 ^b^	124.03 ± 3.97 ^c^
Cr (mg/dL)	0.63 ± 0.06 ^b^	0.62 ± 0.04 ^b^	0.59 ± 0.07 ^b^	1.29 ± 0.10 ^a^	0.84 ± 0.09 ^b^	0.76 ± 0.08 ^b^
Urea (mg/dL)	27.71 ± 1.63 ^d^	26.32 ± 2.55 ^d^	25.41 ± 1.83 ^d^	59.32 ± 2.11 ^a^	52.13 ± 3.03 ^b^	35.27 ± 2.37 ^c^
ALP (U/L)	84.74 ± 3.78 ^cd^	82.12 ± 2.65 ^cd^	79.49 ± 3.44 ^d^	157.41 ± 5.12 ^a^	123.26 ± 4.16 ^b^	93.34 ± 5.02 ^c^
ALT (U/L)	39.64 ± 2.30 ^c^	36.63 ± 2.76 ^c^	35.24 ± 3.63 ^c^	89.94 ± 4.16 ^a^	63.37 ± 3.19 ^b^	42.32 ± 3.90 ^c^
AST (U/L)	67.41 ± 2.53 ^c^	65.27 ± 2.37 ^c^	64.46 ± 1.93 ^c^	117.12 ± 3.68 ^a^	96.32 ± 5.32 ^b^	74.52 ± 4.12 ^c^

TP: Total protein; Glo: Globulin; Alb: Albumin; TG: Triacylglycerides; TC: Total cholesterol; Cr: Creatinine; ALP: Alkaline phosphatase; ALT: Alanine transaminase; AST: Aspartate transaminase. RT: Rutin (25 mg/kg body weight); RT-PNPs: Rutin-loaded nano-phytosomes (25 mg/kg body weight); EAC: Inoculated with Ehrlich Ascites Carcinoma (EAC) cells (0.2 mL); EAC/RT: Rutin (25 mg/kg body weight) + EAC cells (0.2 mL); EAC/RT-PNPs: Rutin-loaded nano-phytosomes (25 mg/kg body weight) + EAC cells (0.2 mL). Within the same row, values bearing different superscript letters (a, b, c, d) differ significantly at *p* < 0.05. Values are presented as mean ± SE.

**Table 4:** Primer Oligonucleotide Sequences Used in Quantitative Real-Time PCR.

Gene	Sequences (5′-3′)	Length (bp)
*TP53*	F: CCCCTGTCATCTTTTGTCCCT R: AGCTGGCAGAATAGCTTATTGAG	137
*Bcl-2*	F: CATCGCCCTGTGGATGACTG R: GGCCATATAGTTCCACAAAGGC	95
*Bax*	F: GTCTCCGGCGAATTGGAGAT R: ACCCGGAAGAAGACCTCTCG	100
*GAPDH*	F: GTATCGGACGCCTGGTTAC R: CTTGCCGTGGGTAGAGTCAT	128

*TP53*: Tumor Protein 53; *Bcl-2*: B-cell lymphoma 2; *Bax*: Bcl-2-associated X protein; *GAPDH*: Glyceraldehyde-3-phosphate dehydrogenase.

**Table 5:** Grading scale for semi-quantitative assessment of hepatic tissue damage.

Score	Integrated Scoring System for Semi-Quantitative Assessment of Hepatic Lesions
0 (none)	Normal histological architecture was observed.
1 (mild)	Occasional hepatocellular degeneration and necrosis were observed, with minimal to absent inflammatory infiltration and rare vascular congestion.
2 (moderate)	Hepatic tissue displayed moderate vacuolar degeneration, multifocal hepatocyte necrosis, scattered inflammatory cell infiltration, and mild congestion of blood vessels.
3 (severe)	Hepatic tissue exhibited diffuse and severe degenerative changes, extensive hepatocellular necrosis, widespread leukocytic infiltration, and moderate to severe congestion of hepatic vasculature.

**Table 6:** A systematic histopathological grading framework for renal tissue using a semi-quantitative scale.

Score	Degenerative and Necrotic Changes in Renal Tubules	Structural Changes in Glomeruli	Inflammation	Hemorrhage
0	None	None	None	None
1	Histopathological examination revealed degenerative changes in 1 to 2 out of 12 fields analyzed.	Mild glomerular atrophy was confined to 1–2 out of 12 assessed fields.	Histological evaluation revealed mild inflammation in 1 to 2 of the 12 assessed areas.	Histological analysis revealed mild glomerular and interstitial congestion with infrequent hemorrhage in 1 to 2 of the 12 evaluated sections.
2	Histological analysis revealed degeneration with multifocal epithelial sloughing in 3 to 6 out of 12 fields.	Mild to moderate glomerular shrinkage appeared in 3 to 4 of the 12 inspected sections.	Histological examination revealed moderate inflammation in 3 to 4 of the 12 assessed areas.	Histopathological evaluation revealed low-grade interstitial congestion and focal hemorrhagic areas in 3 to 4 out of 12 fields.
3	Histopathological assessment revealed widespread tubular necrosis in 7 to 9 of the 12 examined areas.	Evidence of lamellar fusion was detected in 5 to 6 of the 12 inspected fields.	Histopathological analysis revealed inflammation in 5–7 of the 12 inspected fields.	Histopathological assessment revealed widespread interstitial congestion and hemorrhagic foci in 5 to 6 out of 12 tissue sections.

## Data Availability

The raw datasets generated and analyzed during the current study are not publicly available due to contractual restrictions with the Northern Border University funding body. Data access requests may be formally submitted to the Deanship of Scientific Research (dsr@nbu.edu.sa) for consideration, in accordance with institutional policy.

## Abbreviations

The following abbreviations are used in this manuscript:

ALT	Alanine aminotransferase
ALP	Alkaline phosphatase
AFP	Alpha fetoprotein
AST	Aspartate aminotransferase
Bax	Bcl-2-associated X protein
Bcl-2	B-cell lymphoma 2
CA-125	Cancer antigen 125
CA 15-3	Cancer antigen 15-3
CA 19-9	Cancer antigen 19-9
CEA	Carcinoembryonic antigen
CAT	Catalase
cDNA	Complementary DNA
COX-2	Cyclooxygenase-2
DLS	Dynamic light scattering
EAC	Ehrlich ascites carcinoma
ELFA	Enzyme-linked fluorescent assay
FMO	Fluorescence minus one
GSH	Glutathione (reduced)
GSH-Px	Glutathione peroxidase
GSK-3β	Glycogen synthase kinase-3 beta
H&E	Hematoxylin and eosin
Hb	Hemoglobin
IL-1β	Interleukin-1 beta
IL-6	Interleukin-6
MDA	Malondialdehyde
MCH	Mean corpuscular hemoglobin
MCHC	Mean corpuscular hemoglobin concentration
MCV	Mean corpuscular volume
MFI	Mean fluorescence intensity
MST	Mean survival time
miRNAs	MicroRNAs
NCI	National Cancer Institute
NF-κB	Nuclear factor kappa B
PCV	Packed cell volume
PLT	Platelet count
PDI	Polydispersity index
KCl	Potassium chloride
PCA	Principal component analysis
RBC	Red blood cell
RT	Rutin
RT-PNPs	Rutin-loaded phytosome nanoparticles
SOD	Superoxide dismutase
TBARS	Thiobarbituric acid reactive substances
TC	Total cholesterol
TP	Total protein
TEM	Transmission electron microscopy
TGs	Triglycerides
TNF-α	Tumor necrosis factor-alpha
WBC	White blood cell
